# Topical interventions for preventing hand-foot syndrome resulting from antineoplastic therapy: A scoping review

**DOI:** 10.1590/1980-220X-REEUSP-2023-0107en

**Published:** 2023-11-10

**Authors:** Fernanda Cristina Gialaim Purcino dos Reis, Amanda Gomes de Menêses, Simone Roque Mazoni, Renata Cristina de Campos Pereira Silveira, Paula Elaine Diniz dos Reis, Christiane Inocêncio Vasques

**Affiliations:** 1 Universidade de Brasília, Faculdade de Ciências da Saúde, Departamento de Enfermagem, Brasília, DF, Brazil.; 2 Universidade de São Paulo, Escola de Enfermagem de Ribeirão Preto, Departamento de Enfermagem Fundamental, Ribeirão Preto, SP, Brazil.

**Keywords:** Hand-Foot Syndrome, Oncology Nursing, Nursing Care, Skin Care, Review, Síndrome Mano-Pie, Enfermería Oncológica, Atención de Enfermería, Cuidados de la Piel, Revisión, Síndrome Mão-Pé, Enfermagem Oncológica, Cuidados de Enfermagem, Higiene da Pele, Revisão

## Abstract

**Objective:**

To map topical interventions used to prevent hand-foot syndrome in cancer patients undergoing antineoplastic therapy.

**Method:**

This is a scoping review reported in accordance with the recommendations of PRISMA-ScR (extension for scoping review) and the Joanna Briggs Institute Manual. The searches were carried out in the electronic databases CINAHL, Cochrane CENTRAL, EMBASE, LILACS, LIVIVO, PubMed, Scopus, Web of Science; and gray literature (Google Scholar, Pro-Quest).

**Results:**

The searches resulted in 12,016 references and the final sample consisted of 45 studies. A total of 42 topical interventions were identified, including: moisturizing creams, corticosteroids, acids, mapisal, silymarin, and henna. However, urea was the most cited intervention (62%). As for the presentations of the interventions, they varied among creams, ointments, gels, hydrocolloids, decoctions, patches, powders, oils, and soaps.

**Conclusion:**

The results allowed reviewing topical interventions, with emphasis on the use of urea and moisturizing creams. However, most of the interventions identified in this review require evaluation in future studies for better understanding of their benefits.

## RESUMO

**Objetivo:**

Mapear as intervenções tópicas utilizadas para a prevenção da síndrome mão-pé em pacientes com câncer em terapia antineoplásica.

**Método:**

Trata-se de uma revisão de escopo reportada de acordo com as recomendações do PRISMA-ScR (extensão para revisão de escopo) e o Manual do Instituto Joanna Briggs. As buscas foram realizadas nas bases eletrônicas CINAHL, Cochrane CENTRAL, EMBASE, LILACS, LIVIVO, PubMed, Scopus, Web of Science; e literatura cinzenta (Google Scholar, Pro-Quest).

**Resultados:**

As buscas resultaram em 12.016 referências e a amostra final foi composta por 45 estudos. Um total de 42 intervenções tópicas foram identificadas, dentre elas: cremes hidratantes, corticosteroides, ácidos, mapisal, silimarina e henna. Entretanto, a ureia foi a intervenção mais citada (62%). Quanto às apresentações das intervenções, estas variaram entre cremes, pomadas, géis, hidrocoloides, decocções, adesivos, pós, óleos e sabões.

**Conclusão:**

Os resultados possibilitaram uma recensão das intervenções tópicas, com destaque ao uso da ureia e cremes hidratantes. Todavia, grande parte das intervenções identificadas nesta revisão necessitam ser avaliadas, em estudos futuros, para melhor compreensão dos seus benefícios.

## RESUMEN

**Objetivo:**

Mapear las intervenciones tópicas utilizadas para prevenir el síndrome mano-pie en pacientes con cáncer sometidos a terapia antineoplásica.

**Método:**

Esta es una revisión del alcance reportada de acuerdo con las recomendaciones de PRISMA-ScR (extensión para la revisión del alcance) y el Manual del Instituto Joanna Briggs. Las búsquedas se realizaron en las bases de datos electrónicas CINAHL, Cochrane CENTRAL, EMBASE, LILACS, LIVIVO, PubMed, Scopus, Web of Science; y literatura gris (Google Scholar, Pro-Quest).

**Resultados:**

Las búsquedas resultaron en 12.016 referencias y la muestra final estuvo compuesta por 45 estudios. Se identificaron 42 intervenciones tópicas, entre ellas: cremas humectantes, corticoides, ácidos, mapisal, silimarina y henna. Sin embargo, la urea fue la intervención más citada (62%). En cuanto a las presentaciones de las intervenciones, variaron entre cremas, ungüentos, geles, hidrocoloides, decocciones, parches, polvos, aceites y jabones.

**Conclusión:**

Los resultados permitieron revisar las intervenciones tópicas, con énfasis en el uso de urea y cremas humectantes. Sin embargo, la mayoría de las intervenciones identificadas en esta revisión deben evaluarse en estudios futuros para mejor comprensión de sus beneficios.

## INTRODUCTION

Hand-foot syndrome (HFS), also known as palmar-plantar erythrodysesthesia, is a dermatological toxicity that affects cancer patients undergoing chemotherapy and/or targeted therapy. HFS was first described in 1974^(1)^ and is characterized by paresthesia, tingling in the palms of the hands, fingers and soles of the feet, which can progress to burning pain, marked erythema with or without edema, skin desquamation, fissures, and ulceration. When it occurs, HFS is generally graded according to the symptoms and signs presented by the patient. According to the CTCAE^(2)^ (Common Terminology Criteria for Adverse Events), their severity can be classified as grade 1 (mild symptoms), grade 2 (moderate symptoms), and grade 3 (severe symptoms).

Although the pathogenesis of HFS has not yet been completely elucidated, one of the theories states that it may be caused by the accumulation of chemotherapy in the eccrine glands, which are more numerous in the palms of the hands and soles of the feet, which can cause metaplasia and focal necrosis of the eccrine duct epithelium^(3)^. Studies indicate that HFS has a high incidence among patients undergoing chemotherapy, ranging from 2% to 60%^(4)^, being even higher among patients who use the antineoplastic capecitabine (47 to 71%)^(5)^. The clinical relevance of HFS is mainly related to the impact on patients’ quality of life. Symptoms and signs, such as pain and discomfort in the hands, can limit activities of daily living such as walking, holding objects, and performing simple tasks. The presence of skin fissures and lesions can impair self-care, such as personal hygiene. In some cases, infections may occur as a result of loss of skin integrity. Symptoms severity is related to the accumulation of chemotherapy doses, and may worsen with each cycle^(6)^. Thus, the development of HFS may interfere with treatment, as dose reduction or interruption of chemotherapy may be necessary to improve symptoms^(7,8)^.

Before starting treatment, the patient must be guided by the nursing team regarding the early identification of symptoms and signs of HFS and about preventive measures, aiming at reducing friction on the skin in the region of the palms of the hands and soles of the feet, such as wearing looser clothing and shoes, avoiding exposure to excessive heat and maintaining hydration of hands and feet by applying moisturizers and emollients twice a day^(9)^.

However, several topical interventions have been evaluated to prevent HFS. The most studied intervention so far is the application of a urea-based cream to the palms of the hands and soles of the feet, which has been shown to be a safe and effective strategy to prevent the occurrence and development of higher degrees of HFS^(10)^. The keratolytic potential of urea reduces hands and feet hyperkeratosis, common in these patients, and helps hydrate and smooth the skin. Other interventions, however, have also been evaluated, such as topical pyridoxine, which is recommended for patients with HFS, given its similarity with the symptoms of vitamin B6 deficiency. Nevertheless, studies evaluating the use of pyridoxine did not identify a reduction in the incidence of HFS in patients who used this intervention^(5,11)^. Furthermore, the use of corticosteroids has also been investigated, as their action reduces inflammation and pain^(6)^.

Several studies have been carried out to evaluate and identify interventions that are effective in preventing HFS and some systematic reviews that evaluated interventions for preventing HFS have been identified^(3,12,13,14,15,16,17,18)^; however, such reviews address specific interventions, such as urea-based cream, pyridoxine, and moisturizing/emollient cream. It is known, however, that other interventions have also been described in the literature for HFS prevention, such as herbal medicines, topical corticosteroids, among others; nonetheless, these interventions have not yet been analyzed by systematic reviews published to date. However, the systematic reviews already published are restricted to evaluating specific interventions and, therefore, their results do not allow observing the universe of existing interventions that have been evaluated for the prevention of HFS. Thus, there is a need to provide a summary of the available evidence regarding topical interventions, as well as identify any gaps in the existing literature.

Therefore, this scoping review aims to map the topical interventions used to prevent HFS in cancer patients undergoing antineoplastic therapy, such as chemotherapy/targeted therapy.

## METHOD

### Protocol and Registration

This scope review was carried out in accordance with the methodology proposed by the Joanna Briggs Institute (JBI)^(19)^ and reported in accordance with the guide *“Preferred Reporting Items for Systematic Reviews and Meta-Analyses for Scoping Reviews”* (PRISMA-ScR)^(20)^. The protocol for this review was registered on the Open Science Framework (OSF) platform^(21)^ under registration number DOI 10.17605/OSF.IO/Y9SB4^(22)^.

### Selection Criteria

This review sought to answer the following guiding question: “What topical interventions are used to prevent hand-foot syndrome in adult cancer patients undergoing chemotherapy/targeted therapy?” This question was formulated based on the PCC strategy^(23)^, where P (Population): adult patients with cancer; C (Concept): topical interventions used to prevent hand-­foot syndrome; and C (Context): chemotherapy/target therapy.

The selection criteria were established based on the guiding question, from the PCC strategy. The following types of studies were included: a) regarding population – studies carried out on cancer patients over 18 years of age; b) regarding the concept – studies that evaluated topical interventions to prevent HFS; c) regarding context – studies evaluating patients undergoing chemotherapy/targeted therapy in an inpatient or outpatient setting; d) regarding design – randomized controlled clinical trials; non-randomized controlled clinical trials; prospective and retrospective cohort studies; case-control studies; analytical cross-sectional studies; case series; reviews and guidelines; b) regarding the type of publication – articles published in journals, dissertations, theses, and monographs. Only studies published in the Latin-Roman alphabet were included. No restriction was used in relation to the publication period.

On the other hand, the following studies were excluded: those evaluating topical interventions for the prevention of HFS in pediatric cancer patients undergoing chemotherapy/targeted therapy, in an inpatient or outpatient setting; studies evaluating non-topical interventions for the prevention of HFS in cancer patients undergoing chemotherapy/targeted therapy; studies evaluating topical interventions for the treatment of HFS in patients undergoing chemotherapy/targeted therapy (HFS classification equal to or greater than grade II); studies evaluating the use of topical interventions for the prevention of HFS in patients undergoing therapies other than chemotherapy/targeted therapy; studies that reported the initiation of topical therapy to prevent HFS after the third administration of chemotherapy/targeted therapy; case reports, letters, conference abstracts, expert opinions, book chapters, research protocols, and pre-clinical studies. The types of publication mentioned above were excluded as they would not allow, due to their characteristics, to identify the information that would meet the objective of this review. Studies published in languages that do not use the Latin-Roman alphabet were also excluded.

### Information Sources

The searches were carried out in the following electronic databases: CINAHL (Cumulative Index to Nursing and Allied Health); Cochrane CENTRAL; BASIS; LILACS (Latin American and Caribbean Literature in Health Sciences); LIVIVE; PubMed; Scopus and Web of Science Core Collection. Furthermore, the search was carried out in gray literature and accessed through the following databases: Google Scholar and Pro-Quest Theses and Dissertations. Additional searches were carried out in the reference lists of included studies. The searches were carried out on July 3, 2022 and updated on February 6, 2023.

### Search Strategy

The search strategy was developed based on controlled and uncontrolled descriptors, present in the thesauruses of MeSH, DeCS, Cinahl and Emtree Titles, as well as keywords identified in the text and titles of potentially eligible studies. Once the terms referring to the PCC acronym were selected, tests were carried out in PubMed and CINAHL, with registration in the information sources. Therefore, the use of the elements of the search strategy referring to P-Population and C-Concept was selected.

**Chart 1:**
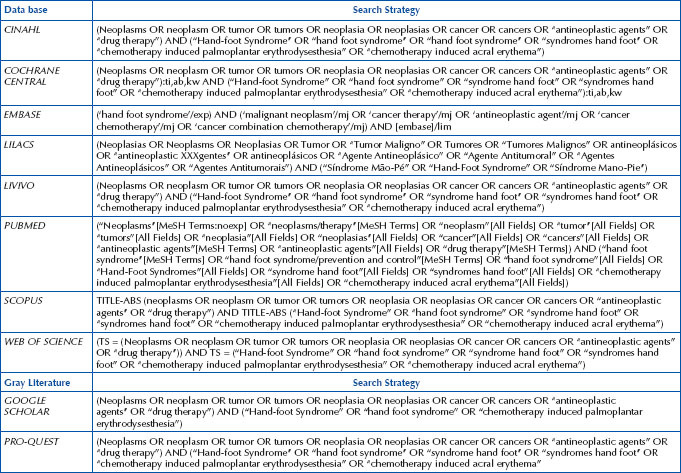
Search strategies applied and adapted to each database – Brasília, DF, Brazil, 2023.

During the selection of the title and full abstract, these elements were considered to find studies relevant to the proposed review. Using the Boolean operator (OR), a single search strategy was created, validated by three researchers, one with expertise in the topic, another with expertise in the topic and method, and a third with expertise in the method. This strategy was adapted to the databases, as well as to the gray literature. It should be noted that to expand the search, a descriptor referring to item “C – Context” of the question was not used. The reference list of included studies was also consulted to find studies that could be included in the review. The search strategy used in each of the electronic databases is available in Chart 1.

### Selection of Sources of Evidence

After finding the studies in the electronic databases, they were exported to the EndNote Web reference manager^(24)^ for duplicate removal and then the reference list was sent to Rayyan^®(25)^, where phase 1 was carried out, in which two reviewers (FCGPR and AGM), blindly, evaluated the titles and abstracts of the identified studies. Those studies that did not meet the inclusion criteria were excluded. In phase 2, the studies were read in full by the same reviewers, also blindly, who again applied the inclusion and exclusion criteria to select the studies. In both phase 1 and phase 2, when the two reviewers did not reach a consensus, the third reviewer with expertise in the topic was called in to evaluate the study and make the final decision.

### Data Collection Procedure

Due to the methodological heterogeneity of the studies, two data collection forms were developed by the authors. One of them, called group I, was used to collect data from intervention studies and systematic reviews, which included the following data: study characteristics (author, year and country of origin of the study, title, design, objective), population characteristics/context (sample, type of cancer, chemotherapy/target therapy, scale used) and characteristics of the intervention/concept (intervention and main results). The other form was used to collect data related to integrative reviews, narratives and guidelines, called group II, which included the following information: study characteristics (author, year, country of origin, title, design, and objective) and characteristics of the intervention/concept (intervention and main results). Two reviewers (F.C.G.P.R. and A.G.M.) carried out a pilot test to evaluate whether the proposed data collection forms allowed collecting all the data necessary for qualitative analysis. Information from studies included in phase 2 was independently extracted using Microsoft Excel spreadsheets^®^.

### Summary of Results

The collected data were presented in a descriptive way, through tables and figures, accompanied by the narrative summary. The data extracted were: year of publication of the studies, country of origin, design, mapping of interventions, studies main results, main cancers studied, the chemotherapy protocols used, as well as the most used scales for HFS grading. Analysis categories were created for each of the identified interventions, grouped into six analysis categories, namely: urea, moisturizing creams, acids, anti-inflammatories/antioxidants, herbal medicine, and other interventions, highlighting the characteristics of the studies, main results, recommendations for clinical practice, limitations, and directions for future studies. It should be noted that the mapped topical interventions were described according to their active ingredient and concentration, when presented. Data collected were grouped to reflect main or recurring themes related to the purpose of the review. To summarize the results, the guidelines contained in the Joanna Briggs Institute Evidence Synthesis Manual were followed^(19)^.

### Ethical Aspects

As it was a scoping review, the research was not submitted for consideration to a Research Ethics Committee.

## RESULTS

### Study Selection

**Figure 1:**
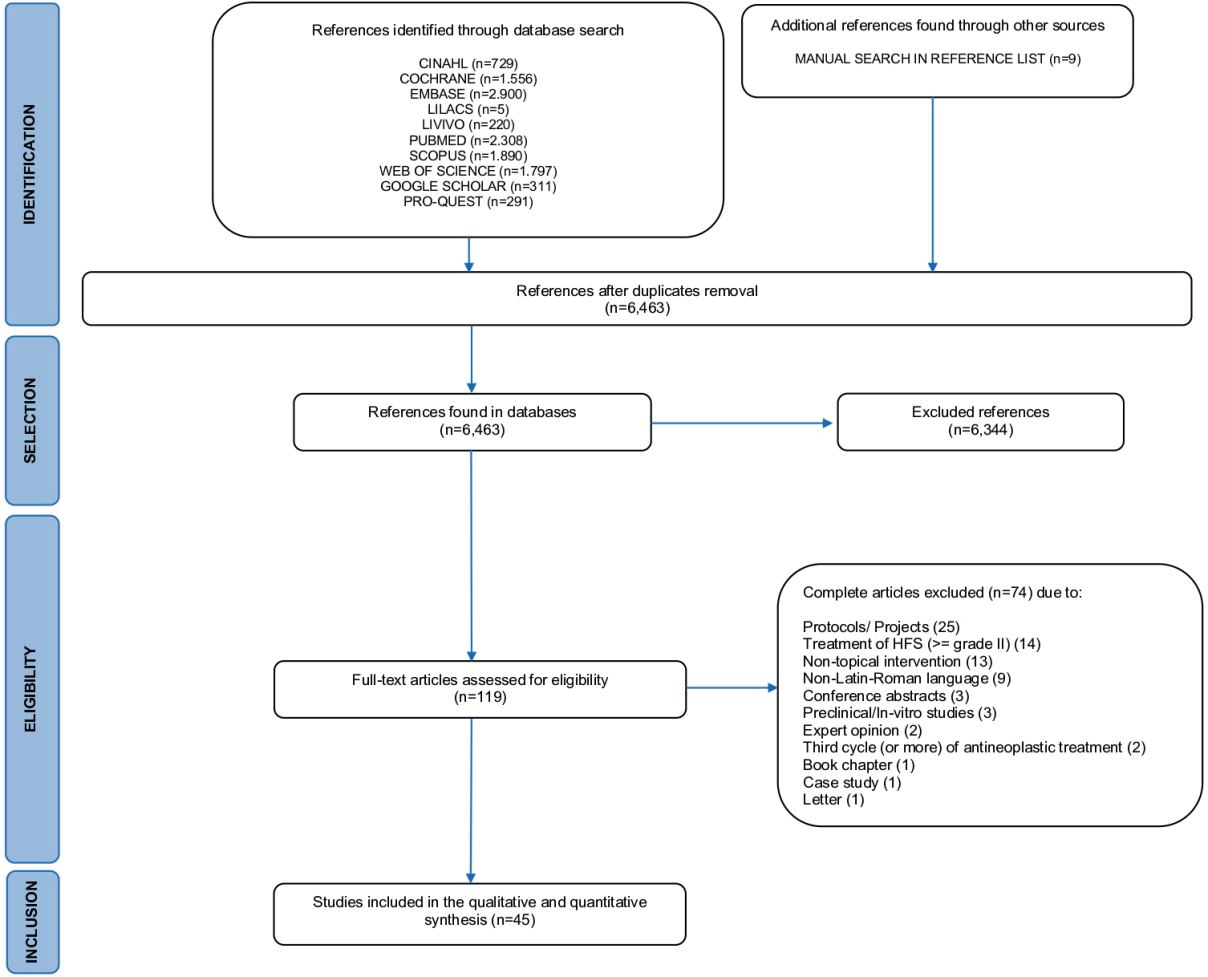
Flowchart of search results in databases and selection criteria. Adapted from PRISMA-ScR^(20)^ – Brasília, DF, Brazil, 2023.

**Chart 2:**
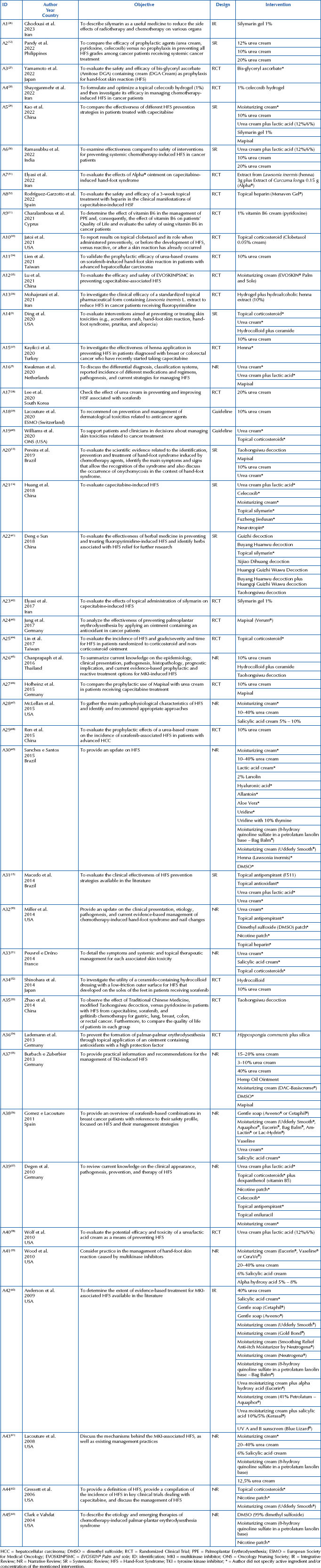
Characteristics of the studies included in the scoping review – Brasília, DF, Brazil, 2023.

Searches in electronic databases resulted in 12,016 references. After removing duplicates, 6,463 references remained, of which 6,344 were excluded in phase 1. One hundred and nineteen studies were selected for full reading and 74 of them were excluded for the reasons listed in Figure 1. The final sample consisted of 45 studies^(3,9,11–15,26–63)^. The characteristics of the individual studies are described in Chart 2. The studies excluded from the review, with their justifications and references, are in the repository *SciELO Data* ^(64)^.

### Characteristics of the Studies

The publication period of the studies ranged from 2004 to 2023, being more concentrated in 2022 (n = 7)^(12,27–32)^. Ten studies were carried out in the United States of America (n = 10)^(3,33,47,50,58–63)^. Regarding the design, twenty studies were randomized clinical trials (RCT) (n = 20)^(27,28,31–38,42–44,46,48,52–54,58)^. The scales used by the RCT to classify HFS were the Common Terminology Criteria for Adverse Events (CTCAE) (n = 17)^(3,12,13,15,27,32–34,36,37,41,43,52–54,58)^; World Health Organization Hand-Foot Syndrome Rating Scale (WHO HFS) (n = 6)^(28,29,31,35,41,42)^. A study^(41)^ cited the Canadian National Cancer Institute Clinical Trials Group (CTG) scale, as well as the two previous scales (WHO HFS and CTCAE). Some studies did not mention the scale used (n = 5)^(14,30,38,44,46)^. Only 4 studies specified the Degree ≤2 of the HFS as inclusion criteria^(27,34,37,52)^ in their methodology.

Among antineoplastic chemotherapy drugs, capecitabine was the most used in the included studies, followed by sorafenib and pegylated liposomal doxorubicin (PLD). The most frequently described types of cancer in the included studies were colon and rectum (n = 11; colon only = 3; intestine only = 1), breast (n = 13), stomach (n = 8) and liver (n = 8). The distribution of all cancer types and chemotherapy protocols mapped in Group I studies, as well as the authors, can be viewed in the repository *SciELO Data* ^(64)^.

**Figure 2:**
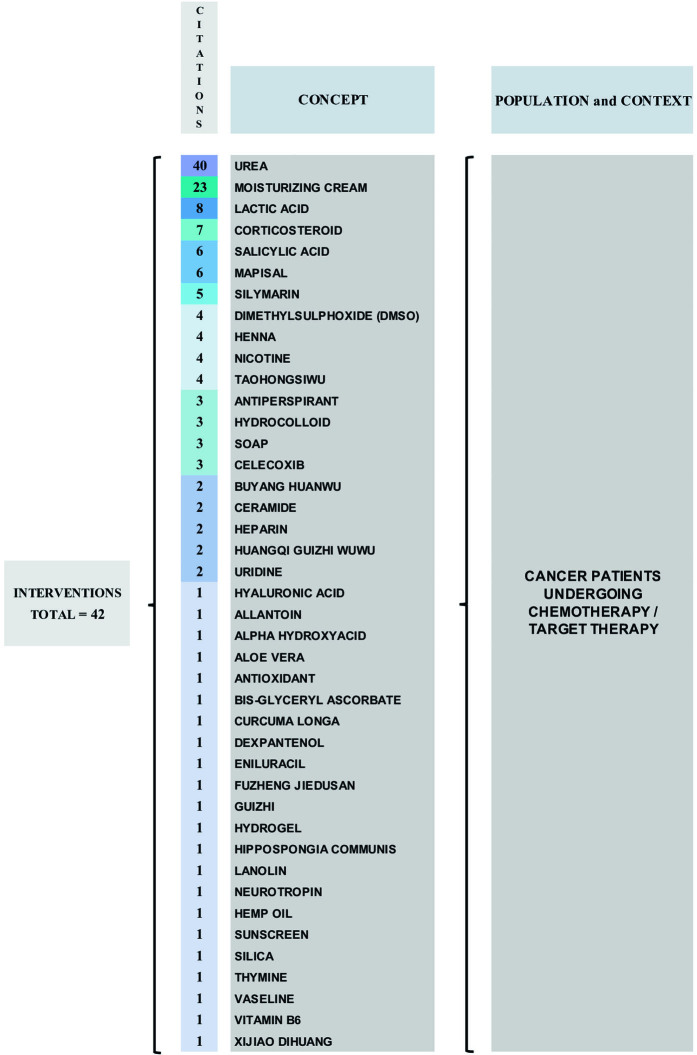
Representation of interventions for preventing HFS cited in the included studies according to the number of citations and following the PCC mnemonic – Brasília, DF, Brazil, 2023.

Regarding the included studies, 42 topical interventions for the prevention of HFS were mapped. Urea was the most investigated intervention (n = 28)^(3,9,12–15,29,30,34,38–40,45–52,55–64)^, followed by moisturizing creams (n = 11)^(14,29,35,47,49,55,56,59–62)^, and corticosteroids (n = 7)^(3,33,40,44,52,57,62)^. It should be noted that, sometimes, the same study evaluated more than one intervention. The topical interventions mapped in the included studies, as well as their number of citations, are described in Figure 2.

### Results of Individual Studies

Urea was the most evaluated intervention in the studies, both in isolated use (n = 28)^(3,9,12–15,29,30,34,38–40,46–52,56–62)^ and combined with lactic acid^(9,14,15,29,30,57,58)^, 5% salicylic acid^(60)^, and alpha hydroxy acid^(60)^. Various concentrations of urea were evaluated in studies, namely: 3%^(52)^, 10%^(3,12,13,29,30,34,39,45–49,52,55)^, 12%^(12)^, 12.5%^(62)^, 15%^(56)^, 20%^(12,30,52,59,61)^ and 40%^(47,49,55,59–61)^; however, some studies did not mention the concentration used^(3,9,13,15,40,50,51,56)^. In some studies, patients were instructed to apply urea cream twice a day^(34.38)^, other studies have recommended applying three times a day^(46,48,52)^. The patients used the urea cream for a period varying from four^(52)^ to twelve weeks^(38,48)^.

The use of moisturizing cream was the second most evaluated intervention in studies (n = 11)^(14,29,35,47,49,55,56,59–62)^. Several moisturizing creams, from the most varied commercial brands, were mentioned, such as: EVOSKIN moisturizing cream^®(29.35)^; Bag Balm^®^ (8-hydroxy quinoline sulfate in petrolatum lanolin base)^(49,56,60,62,63)^; UdderlySmooth^®(49,56,60,62)^; DAC-Basiscreme^®(57)^; Aquaphor^®^ (petrolatum 41%)^(56,60)^; Eucerin^®(56.59)^; AmLactin^®(56)^; LacHydrin^®(56)^; Vaseline^®(59)^; CeraVe^®(59)^; Soothing Relief Anti-itch Moisturizer By Neutrogena^®(60)^; Neutrogena foot cream^®(60)^; and the Gold Bond^®(60)^. Some studies only cited “moisturizing cream” as an intervention, without mentioning the active ingredient or commercial name^(14,47,49,61)^.

Some acid-based creams were cited as topical interventions, including creams based on salicylic acid in concentrations of 5% and 10%^(47)^, and 6%^(59.61)^. Some studies that evaluated salicylic acid-based creams did not describe the concentration used^(51,56)^. The lactic acid-based cream shall be highlighted, as, despite being present as one of the most cited interventions, was described separately in only one study^(52)^; in other citations, it was associated with urea-based cream^(9,14,15,29,30,57,58)^.

The use of topical corticosteroids was mapped in seven studies^(3,33,40,44,51,57,61)^. Among them, the use of clobetasol cream at 0.05%^(33)^ and topical steroids associated with dexpanthenol^(57)^ was observed. The other studies did not inform the type of topical corticosteroid or its concentration^(3,40,44,51,62)^. Three studies cited celocoxib, a non-steroidal anti-inflammatory drug^(14,28,29)^. Studies evaluating the use of antioxidants cited the use of mapisal ointment^(9,13,29,43,46,56)^ or did not describe the antioxidant active ingredient^(15)^.

Topical herbal interventions have been extensively studied. Some studies cited Taohongsiwu, composed of Taoren (*Semen persicae*) 30g, Honghua (*Flos carthami*) 30g, Shudihuang (*Radix rehmanniae praeparata*) 30g, Danggui (*Radix angelica sinensis*) 30g, Chuanxiong (*Rhizoma chuanxiong*) 15g, Baishao (*Radix paeoniae alba*) 15g, Guizhi (*Ramulus cinnamomi*)15g, Chuanniuxi (*Radix cyathulae*) 15g, Gancao (*Radix glycyrrhizae*) 6g and Dazao (*Fructus jujubae*) three pieces^(13,41,45,53)^. Other compounds were Guizhi, *Buyang huanwu*, *Xijiao dihuang*, *Huangqi guizhi wuwu,* and Taohongsiwu^(41)^. Other studies also mentioned silymarin without the concentration mentioned^(14.41)^ and 1% silymarin^(26,29,42)^; *Fuzheng jiedusan* ^(14)^; extract of *Lawsonia inermis* (henna) 3g plus *Curcuma longa* 0.15g^(31)^ extract ; hydrogel plus 10% hydroalcoholic henna extract^(36)^; *Lawsonia inermis* (henna)^(37,49)^; allantoin^(49)^; Aloe vera^(49)^; *Hippospongia communis* plus silica oil in water^(54)^; and hemp oil^(55)^.

Other topical interventions were mapped, such as hydrocolloid dressing containing ceramide^(3.45)^; topical neurotropin^(14)^; bis-glyceryl ascorbate^(27)^; topical heparin^(32.50)^; 1% topical pyridoxine (vitamin B6)^(11)^; hyaluronic acid^(49)^; topical lanolin^(49)^; isolated topical^(49,57)^ uridine, or uridine associated with 10% thymine^(49)^; hydrocolloid dressing^(52)^; topical DMSO (dimethyl sulfoxide)^(49,55,63)^; vaseline^(56)^; topical eniluracil^(57)^; as well as the use of UV A and B sunscreen (Blue Lizard^®^)^(60)^.

In addition to the above-mentioned interventions, topical antiperspirants were also mentioned, such as F511^(15.57)^; however, one of the studies that mentioned the use of antiperspirants did not mention the active ingredient that made up the product^(50)^. The use of dimethyl sulfoxide (DMSO)^(50)^ and nicotine^(50,57,62,63)^ patches, as well as soaps, such as Aveeno^®^ and/or Cetaphil^®(56.60)^ were also evaluated as interventions to prevent HFS.

In short, of the 45 studies surveyed, less than half of them were RCTs (44%) (n = 20). Among the RCTs, 30% evaluated the use of herbal medicines (n = 6) and 25% evaluated the use of urea (n = 5). It was also observed that the studies included in this review were published in the last 20 years, ranging from 2004 to 2023, with a higher prevalence in 2022 (n = 7). Regarding the region of origin of the studies, although the number of countries where the studies were conducted was greater in the East (n = 10), there was a greater, albeit discreet, volume of publications in Western countries, with 51% of the studies carried out in this region. Of the cancers cited, 24.4% were of the intestine/colon/rectum, followed by 20.3% breast cancer and 14% esophagogastric cancer. Regarding the type of chemotherapy, 71% of the studies that cited antineoplastic protocols mentioned capecitabine (n = 20). Of the total interventions mapped, 42 topical interventions were identified to prevent hand-foot syndrome, with 62% of the studies mentioning urea (n = 28) (alone or combined), regardless of the type of cancer and chemotherapy protocol. As for the presentations of the interventions, they varied among creams, ointments, gels, hydrocolloids, decoctions, patches, powders, oils, and soaps.

## DISCUSSION

This scoping review aimed to map topical interventions to prevent HFS. Forty-two interventions were identified and the most cited intervention was urea-based cream. Urea is a polar and hygroscopic molecule produced endogenously by the human body and naturally found in the skin. It originates from the metabolism of proteins and other organic nitrogen compounds excreted in urine and sweat, and has the effect of softening hyperkeratosis and reducing epidermal thickness^(65)^.

The results of a meta-analysis^(18)^ with 1,387 patients, which evaluated the effectiveness of urea cream in preventing and treating HFS, found that patients who received the urea cream intervention had a significantly lower incidence of grade 2 or higher HFS (risk ratio, 0.72; 95% confidence interval, 0.58–0.90) and lower incidence of HFS of any grade (risk ratio, 0.79; 95% confidence interval, 0.58–1.08) when compared with patients who did not receive urea cream intervention. It was concluded that urea cream is a safe and viable topical intervention for preventing HFS.

In another meta-analysis^(12)^ with 2,814 patients, which evaluated prophylactic strategies for HFS, both topical and nontopical, it was observed that urea cream (topical route) (OR 0.48, 95% CI 0.39–0.60, p < 0.00001) and celecoxib (oral) (OR 0.52, 95% CI 0.32–0.85, p = 0.009) showed a significant risk reduction, with celecoxib being more effective in preventing capecitabine-induced HFS in all grades (50.5% vs 65%, p = 0.05), while urea cream showed more benefits in preventing sorafenib-induced moderate to severe HFS (54.9% vs 71.4%, P < 0.00001). However, studies investigating the ideal dosage of celecoxib and urea cream are recommended.

On the other hand, two recently published network metaanalyses^(29,30)^, which sought to evaluate the best intervention for preventing HFS, and which analyzed urea, were not in line with previous information. The first study^(29)^ analyzed, as topical interventions, moisturizing cream, 10% urea cream, urea and lactic acid cream (12%/6%), 1% silymarin gel and mapisal, and as a result, showed that topical silymarin has the best performance in preventing capecitabine-induced HFS (OR: 0.08; 95% CI: 0.01–0.71). The second^(30)^ one analyzed, as topical interventions, urea and lactic acid cream (12%/6%), 10% urea cream, and 20% urea cream; however, the interventions investigated did not demonstrate a significant role in preventing HFS.

Moisturizing creams were widely used in many studies identified by this scoping review, but only one of them was an RCT. This study^(35)^ evaluated 51 patients who were instructed to apply the cream to their hands and feet twice a day, until the end of treatment with capecitabine, versus the use of saline solution. The total incidence of HFS in the group that used moisturizing cream was lower than that in the group that used saline solution (56.8% vs. 75.9%, P = 0.006). The incidence of Grade 1–2 HFS was not statistically significant between the two groups (26/51 vs. 32/54, 52.0% vs. 59.2%, P = 0.194).

Another intervention mapped out in this review was the use of topical corticosteroids, with local anti-inflammatory action. The result of one of the studies^(44)^ found demonstrated a statistically significant difference between HFS overall score among patients who received corticosteroid cream and those who received non-corticosteroid cream (0.83 vs. 1.26, p = 0.031). There was also a statistically significant difference between patients who received the corticosteroid cream intervention and those patients who did not receive any intervention (0.83 vs. 1.24, p = 0.038). The time to onset of HFS was longer in the group that used corticosteroid cream (41 days) when compared to the group that used non-corticosteroid cream (22 days) and in the control group without intervention (21 days). Although the study did not identify which corticosteroid was used or its concentration, it was concluded that the use of topical corticosteroids can reduce the severity and incidence of HFS.

The results brought by another study^(33)^ included in this review demonstrated that preventive therapy with clobetasol, a high-potency topical corticosteroid, is associated with lower rates of regorafenib-induced HFS. During the second cycle of regorafenib, the frequency of HFS was 30% for grade 1, 8% for grade 2, and 3% for grade 3, with the use of preventive clobetasol. The study also evaluated the use of clobetasol after the development of HFS and the frequency of HFS was 43% for grade 1, 18% for grade 2, and 7% for grade 3 (p = 0.12).

In addition to the above-mentioned interventions, it is worth highlighting that many studies have included the use of plants in the composition of products as alternatives for the prevention of HFS. Among the plants used, henna stands out, a dye extracted from dried leaves and branches of *Lawsonia inermis*, with antioxidant and immunomodulatory effects^(66,67,68)^; curcumin, the main component of turmeric, known for its anti-inflammatory and antioxidant activities, for preventing the activation of the biosynthesis of prostaglandins and c-Jun/AP-1, protein kinases and expression of COX-2^(69,70)^; and silymarin, a member of the *Asteraceae* family, which acts as an antioxidant and inhibitor of lipid peroxidation activity, has an immunomodulatory effect, increasing the proliferation of lymphocytes, interferon gamma (IFN-*γ*), secretion of IL-4, and IL-10 by lymphocytes and suppression of the activation of T cells, by affecting the NF-kB pathway^(28,71,72,73)^.

Regarding HFS staging, it is known that it is essential to classify it using a validated and standardized instrument, so that it is possible to compare the progression and effect of the interventions studied. Therefore, different instruments have been used to grade the severity of HFS, but the CTCAE classification^(2)^ (Common Terminology Criteria for Adverse Events), published by the National Cancer Institute of the United States of America, is the best-known scale and was the most used by the studies included in this review.

The CTCAE^(2)^ (version 5.0 of 2017) classifies HFS into three degrees of severity, in which Grade 1 represents minimal changes in the skin or dermatitis, without pain; Grade 2 indicates changes in the skin, with pain, limiting instrumental Activity of Daily Living (ADL); and Grade 3 indicates severe skin changes, with pain, limiting self-care and ADL. Two other scales were used by some studies, namely: World Health Organization scale (WHO HFS), which has four grades of HFS classification^(74)^ and the Canadian National Cancer Institute Clinical Trials Group (CTG-NCIC) scale, which classifies HFS into three grades^(75)^.

Through this scoping review, it was possible to map a significant quantity of interventions that have been investigated for HFS prevention. However, a gap identified in the literature concerns the still incipient knowledge about the pathophysiology of HFS, which hinders the finding of pharmacological mechanisms that can prevent its occurrence. Thus, what can be seen is the search for some effective intervention through “trial and error”. However, studies seek to find a mechanism of action for the development of HFS or even genetic markers or predictors that can predetermine its incidence and/or severity and determine new strategies to improve patients’ quality of life. Understanding this development mechanism is essential for carrying out research into topical therapies for the prevention of HFS, to develop studies with clinical relevance, and avoid studies with little statistical significance or even therapeutic futility.

As already postulated, capecitabine is the main chemotherapy drug associated with the development of HFS. Studies show that most patients treated with capecitabine develop HFS. Capecitabine is a prodrug of 5-fluorouracil, administered orally and commonly used in some solid tumors, such as colorectal, gastric, and breast cancer. At a cellular level, capecitabine toxicity induces the death of keratinocytes and reduces the stratum corneum present in this condition^(34,46)^. A recent study retrospectively analyzed the medical records of 165 patients treated with capecitabine and identified significant predictors for the development of HFS, such as concomitant use of a renin-angiotensin system inhibitor medication, high body surface area, and albuminemia^(76)^.

Another study^(77)^, whose sample consisted of patients with advanced colon cancer treated with capecitabine and oxaliplatin (XELOX), investigated the rs6783836 variant in ST6GAL1 (ST6 β-galactoside α-2,6-sialyltransferase), a gene that plays a role in inflammation and development of type 2 diabetes, and concluded that the gene was associated with the development of HFS, showing to be a promising biomarker of the syndrome^(77)^. Furthermore, an important study^(78)^ revealed a novel mechanism of individual genetic susceptibility to capecitabine-associated HFS, with implications for clinically relevant risk prediction. To this end, an extreme phenotype test was performed for a genome-wide association study in 166 patients, which revealed that the skin of the patient with severe HFS showed low levels of R-cadherin and Involucrin (proteins that are essential for the structure and function of the skin barrier) before treatment with capecitabine. Studies evaluating risk prediction through individualized genomic mapping can contribute to the selection of more powerful interventions indicated early for this type of patient.

Additionally, this review showed that pegylated liposomal doxorubicin (PLD) appears to be associated with the emergence of HFS, being the subject of studies whose objective was to search for the cause of this syndrome. Corroborating this, there was a study^(79)^ that, through histological analysis of HFS *in vitro* and in animal models, found that PLD induces severe tissue damage, including the destruction of collagen fibers and the induction of severe inflammation and epidermal cells apoptosis. Due to this inflammation and the sustained release of PLD, reactive oxygen species (ROS) were generated, which are unstable and extremely reactive molecules, capable of transforming other molecules with which they collide, causing oxidative damage to keratinocytes. Therefore, the study concluded that the generation of ROS was identified as a crucial factor in the development of HFS and could be used as a potential therapeutic target for future studies. Therefore, antioxidant interventions can be a good option for patients on PLD use.

The role of nurses is essential for the management of cancer patients undergoing chemotherapy and targeted therapy, especially those at risk of developing HFS. A study^(80)^ demonstrates that patients who were in contact with oncologist nurses and followed their recommendations had a fifty-fold reduction in the risk of developing grade 2 or 3 HFS. Among the nursing care for managing HFS, the importance of a systematic evaluation by nurses before and during systemic antineoplastic treatment stands out, including: evaluation of the chemotherapy protocol, number of expected cycles, monitoring of laboratory tests, application of a scale of pain, assessment of mobility, nutritional status, psychological status, local assessment of the hands and feet (with assessment of the radial, brachial, dorsal artery of the foot and posterior tibial artery) and, if possible, prescription of interventions to prevent HFS. If there is already an injury, assess: type of wound; possible causes; location, size and depth; characteristic of the wound bed and edges; presence of exudate and its characteristics; peri-lesion skin characteristic; possible therapies^(81)^.

Nurses can also use strategies to identify possible risk factors for the development of HFS. A tool^(82)^ of clinical scoring, using machine learning, was validated to prevent severe HFS in patients with hepatocellular carcinoma on sorafenib. The results showed that being female, having high hemoglobin, and low bilirubin were factors that showed high discrimination in predicting the risk of HFS. Such a tool can allow the monitoring of patients who are at high risk for developing HFS and the early identification of symptoms AND SIGNS.

Therefore, using effective means of health education that improve the communication of symptoms and signs by patients is vital for managing toxicities caused by chemotherapy and improving the quality of life of patients who develop HFS.

### Study Limitations

The results of this review may have been limited for a few reasons. The lack of information about the active ingredients and concentrations, especially in studies including the use of topical anti-inflammatories/antioxidants and moisturizing creams. The lack of information about the chemotherapy cycle may have led to the exclusion of some studies, since one of the inclusion criteria for this review was that the intervention should not be started after the second cycle of chemotherapy. Finally, another important limitation was the exclusion of studies published in non-Latin-Roman languages. Such limitations may have made the inclusion of some studies unfeasible or hindered a more critical and detailed analysis of the included studies.

### Implications for the Advancement of Scientific Knowledge for the Area of Health and Nursing

The results of this scoping review demonstrate to the scientific community, especially to the nursing team, the topical interventions described in the literature for the prevention of HFS in cancer patients undergoing antineoplastic chemotherapy/target therapy. This study can help researchers identify interventions that can be evaluated for the prevention of HFS, so that the reproduction of studies evaluating interventions that already have their effects well-elucidated in the literature is avoided.

## CONCLUSION

This scoping review mapped 42 topical interventions for HFS prevention in cancer patients undergoing antineoplastic therapy. The results allowed reviewing topical interventions, with emphasis on the use of urea and moisturizing creams as the most studied interventions. However, most of the interventions identified in this review require evaluation in future studies for better understanding of their benefits.
